# 1-Methyl-2-[(*E*)-2,4,5-trimeth­oxy­styr­yl]pyridinium 4-meth­oxy­benzene­sulfonate monohydrate

**DOI:** 10.1107/S1600536811008610

**Published:** 2011-03-12

**Authors:** Hoong-Kun Fun, Charoensak Mueangkeaw, Pumsak Ruanwas, Suchada Chantrapromma

**Affiliations:** a X-ray Crystallography Unit, School of Physics, Universiti Sains Malaysia, 11800 USM, Penang, Malaysia; b Crystal Materials Research Unit, Department of Chemistry, Faculty of Science, Prince of Songkla University, Hat-Yai, Songkhla 90112, Thailand

## Abstract

In the title compound, C_17_H_20_NO_3_^+^·C_7_H_7_O_4_S^−^·H_2_O, the cation exists in an *E* configuration with respect to the C=C bond and is twisted with a dihedral angle of 17.81 (8)° between the pyridinium and benzene rings. The benzene ring of the anion is almost parallel to the pyridinium ring [dihedral angle = 3.45 (9)°], whereas it is inclined to the benzene ring of the cation [dihedral angle = 17.62 (8)°]. The crystal structure is stabilized by O—H⋯O hydrogen bonds and weak C—H⋯O inter­actions which link the cations, anions and water mol­ecules into chains along the *a* axis. π–π inter­actions with centroid–centroid distances of 3.7751 (9) and 3.7920 (11) Å are also observed.

## Related literature

For bond-length data, see: Allen *et al.* (1987 ▶). For background to the non-linear optical properties and applications of pyridinium and quinolinium derivatives, see: Chanawanno *et al.* (2010 ▶), Chantrapromma *et al.* (2010 ▶); Fun *et al.* (2009 ▶); Ruanwas *et al.* (2010 ▶); Williams (1984 ▶). For related structures, see, Chantrapromma *et al.* (2007 ▶); Mueangkeaw *et al.* (2010 ▶). For the stability of the temperature controller used in the data collection, see Cosier & Glazer, (1986 ▶).
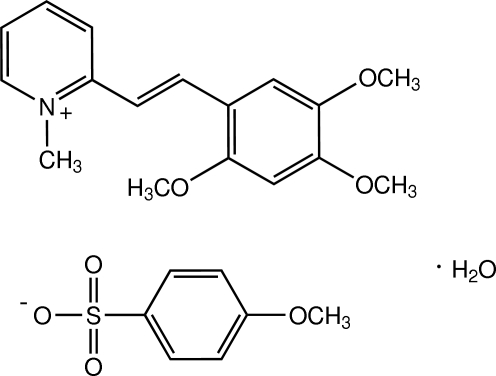


## Experimental

### 

#### Crystal data

C_17_H_20_NO_3_^+^·C_7_H_7_O_4_S^−^·H_2_O
*M* *_r_* = 491.55
Triclinic, 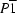

*a* = 6.8463 (4) Å
*b* = 10.8855 (5) Å
*c* = 15.8137 (8) Å
α = 83.950 (2)°
β = 81.355 (2)°
γ = 81.140 (2)°
*V* = 1147.14 (10) Å^3^
*Z* = 2
Mo *K*α radiation
μ = 0.19 mm^−1^
*T* = 100 K
0.40 × 0.08 × 0.06 mm

#### Data collection

Bruker APEXII CCD area-detector diffractometer
Absorption correction: multi-scan (*SADABS*; Bruker, 2005 ▶) *T*_min_ = 0.927, *T*_max_ = 0.989
20386 measured reflections
5219 independent reflections
4534 reflections with *I* > 2σ(*I*)
*R*_int_ = 0.044

#### Refinement

*R*[*F* ^2^ > 2σ(*F* ^2^)] = 0.045
*wR*(*F* ^2^) = 0.124
*S* = 1.05
5219 reflections
320 parameters
H atoms treated by a mixture of independent and constrained refinement
Δρ_max_ = 0.54 e Å^−3^
Δρ_min_ = −0.56 e Å^−3^

### 

Data collection: *APEX2* (Bruker, 2005 ▶); cell refinement: *SAINT* (Bruker, 2005 ▶); data reduction: *SAINT*; program(s) used to solve structure: *SHELXTL* (Sheldrick, 2008 ▶); program(s) used to refine structure: *SHELXTL*; molecular graphics: *SHELXTL*; software used to prepare material for publication: *SHELXTL* and *PLATON* (Spek, 2009 ▶).

## Supplementary Material

Crystal structure: contains datablocks global, I. DOI: 10.1107/S1600536811008610/rz2563sup1.cif

Structure factors: contains datablocks I. DOI: 10.1107/S1600536811008610/rz2563Isup2.hkl

Additional supplementary materials:  crystallographic information; 3D view; checkCIF report

## Figures and Tables

**Table 1:** Hydrogen-bond geometry (Å, °)

*D*—H⋯*A*	*D*—H	H⋯*A*	*D*⋯*A*	*D*—H⋯*A*
O1*W*—H2*W*1⋯O6	0.80 (3)	2.08 (3)	2.8703 (19)	175 (3)
O1*W*—H1*W*1⋯O5^i^	0.85 (3)	1.98 (3)	2.8233 (19)	173 (2)
C9—H9*A*⋯O4	0.93	2.53	3.445 (2)	167
C14—H14*A*⋯O1*W*^ii^	0.96	2.32	3.262 (2)	168
C14—H14*C*⋯O1^iii^	0.96	2.54	3.487 (2)	168
C16—H16*A*⋯O7^iv^	0.96	2.53	3.388 (2)	149
C16—H16*B*⋯O5^iii^	0.96	2.54	3.408 (2)	150
C16—H16*C*⋯O6^v^	0.96	2.44	3.371 (2)	163
C17—H17*C*⋯O4^iii^	0.96	2.47	3.419 (2)	168
C18—H18*A*⋯O1*W*^vi^	0.93	2.43	3.354 (2)	171
C22—H22*A*⋯O2^iv^	0.93	2.36	3.281 (2)	170

Symmetry codes: (i) 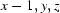; (ii) 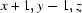; (iii) 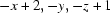; (iv) 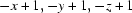; (v) 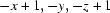; (vi) 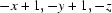.

**Fig. 1.**
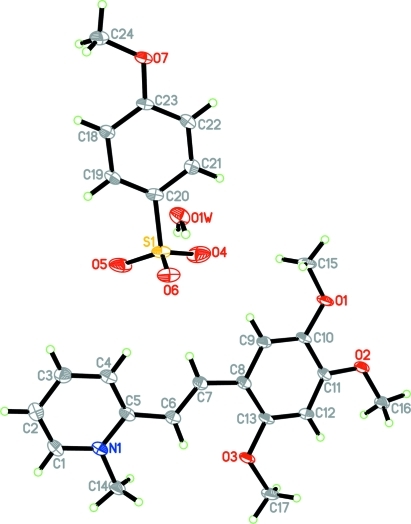
The asymmetric unit of the title compound, with 50% probability displacement ellipsoids.

**Fig. 2.**
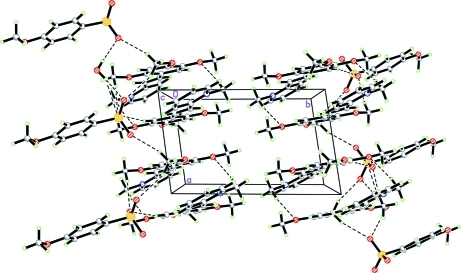
The crystal packing of the title compound viewed approximately down the c-axis. Hydrogen bonds are shown as dashed lines.

## supplementary crystallographic information

### Comment

Within the frame of our on-going research on non-linear optic (NLO) materials and antibacterial compounds, we have synthesized several pyridinium and quinolinium derivatives, and their NLO properties and antibacterial activities have been reported (Chanawanno *et al.*, 2010; Chantrapromma *et al.*, 2007; Fun *et al.*, 2009; Ruanwas *et al.*, 2010). As part of this research the title pyridinium derivative, (I), was synthesized and its crystal structure is herein reported. The title compound crystallizes in the centrosymmetric triclinic *P*1 space group and therefore it does not exhibit second order NLO properties (Williams, 1984). In addition, (I) was also tested for antibacterial activities against *Bacillus subtilis*, *Enterococcus faecalis*, *Staphylococcus aureus*, methicillin-resistant *Staphylococcus aureus*, vancomycin-resistant *Enterococcus faecalis*, *Pseudomonas aeruginosa*, *Salmonella typhi* and *Shigella sonnei*, and it was found to be inactive.

Fig. 1 shows the asymmetric unit of (I) which consists of a C_17_H_20_NO_3_^+^ cation, a C_7_H_7_O_4_S^-^ anion and a water solvent molecule. The cation exists in the *E* configuration with respect to the C6═C7 double bond [1.352 (2) Å] with the torsion angle C5–C6–C7–C8 = -174.98 (16)°. The cation is twisted with the dihedral angle between the pyridinium and benzene rings of 17.81 (8)°. One of the three methoxy substituent of the 2,4,5-trimethoxyphenyl ring is twisted whereas the other two are essentially co-planar with torsion angles of 6.4 (2), 1.7 (2) and 0.7 (2)° for C15–O1–C10–C9, C16–O2–C11–C12 and C17–O3–C13–C12, respectively. The methyl groups of two methoxy substituents at atoms C11 and C13 point toward whereas at atoms C10 and C11 point away from each other (Fig. 1) due to the steric effect of their positions. In the anion, the methoxy group is co-planar with the benzene ring forming a torsion angle C24–O7–C23–C18 = 1.2 (2)°. The benzene ring of the anion is almost parallel to the pyridinium ring (dihedral angle 3.45 (9)°), whereas it is inclined to the benzene ring of the cation at 17.62 (8)°. The bond lengths of (I) are in normal ranges (Allen *et al.*, 1987) and comparable to those found in related structures (Chantrapromma *et al.*, 2010; Mueangkeaw *et al.*, 2010).

In the crystal packing, the cations are linked to both anions and water molecules by weak C—H···O interactions, and the anions are linked to water molecules by O—H···O hydrogen bonds to form chains along the *a* axis (Table 1, Fig. 2). π–π interactions are observed with centroid-to-centroid distances Cg_1_···Cg_2_^i^ = 3.7920 (11) Å and Cg_3_···Cg_3_^ii^ = 3.775 (9) Å; Cg_1_, Cg_2_ and Cg_3_ are the centroids of the N1/C1–C5, C18–C23 and C8–C13 rings, respectively (symmetry codes: (i) *x*, -1+*y*, *z*; (ii) = 2-*x*, -*y*, 1-*z*).

### Experimental

1-Methyl-2-[(*E*)-2,4,5-trimethoxystyryl]pyridinium iodide (compound A) was prepared according to the previously reported method (Mueangkeaw *et al.*,2010). Silver(I) 4-methoxybenzenesulfonate (compound B) was synthesized by following the previous procedure (Chantrapromma *et al.*,2007). The title compound was prepared by mixing a 1:1 molar ratio of compound A (0.100 g, 0.24 mmol) and compound B (0.071 g, 0.24 mmol) in hot CH_3_OH (50 ml). The mixture immediately yielded a grey precipitate of silver iodide. After stirring the mixture for ca. 30 min, the precipitate was removed and the resulting solution was evaporated yielding an orange viscous oil. Orange needle-shaped single crystals of the title compound suitable for *x*-ray structure determination were recrystallized from DMSO by slow evaporation at room temperature over a few weeks, M.p. 453-454 K.

### Refinement

Water H atoms were located in difference maps and refined isotropically. The remaining H atoms were positioned geometrically and allowed to ride on their parent atoms, with d(C-H) = 0.93 Å for aromatic and CH and 0.96 Å for CH_3_ atoms. The *U*_iso_ values were constrained to be 1.5*U*_eq_ of the carrier atom for methyl H atoms and 1.2*U*_eq_ for the remaining H atoms. A rotating group model was used for the methyl groups. The highest residual electron density peak is located at 0.75 Å from O6 and the deepest hole is located at 0.75 Å from S1.

### Crystal data

**Table:**

C_17_H_20_NO_3_^+^·C_7_H_7_O_4_S^−^·H_2_O	*Z* = 2
*M**_r_* = 491.55	*F*(000) = 520
Triclinic, *P*1	*D*_x_ = 1.423 Mg m^−^^3^
Hall symbol: -P 1	Melting point = 453–454 K
*a* = 6.8463 (4) Å	Mo *K*α radiation, λ = 0.71073 Å
*b* = 10.8855 (5) Å	Cell parameters from 5219 reflections
*c* = 15.8137 (8) Å	θ = 1.9–27.5°
α = 83.950 (2)°	µ = 0.19 mm^−^^1^
β = 81.355 (2)°	*T* = 100 K
γ = 81.140 (2)°	Needle, orange
*V* = 1147.14 (10) Å^3^	0.40 × 0.08 × 0.06 mm

### Data collection

**Table:**

Bruker APEXII CCD area-detector diffractometer	5219 independent reflections
Radiation source: sealed tube	4534 reflections with *I* > 2σ(*I*)
graphite	*R*_int_ = 0.044
φ and ω scans	θ_max_ = 27.5°, θ_min_ = 1.9°
Absorption correction: multi-scan (*SADABS*; Bruker, 2005)	*h* = −8→8
*T*_min_ = 0.927, *T*_max_ = 0.989	*k* = −14→14
20386 measured reflections	*l* = −20→20

### Refinement

**Table:**

Refinement on *F*^2^	Primary atom site location: structure-invariant direct methods
Least-squares matrix: full	Secondary atom site location: difference Fourier map
*R*[*F*^2^ > 2σ(*F*^2^)] = 0.045	Hydrogen site location: inferred from neighbouring sites
*wR*(*F*^2^) = 0.124	H atoms treated by a mixture of independent and constrained refinement
*S* = 1.05	*w* = 1/[σ^2^(*F*_o_^2^) + (0.0633*P*)^2^ + 0.7052*P*] where *P* = (*F*_o_^2^ + 2*F*_c_^2^)/3
5219 reflections	(Δ/σ)_max_ = 0.001
320 parameters	Δρ_max_ = 0.54 e Å^−^^3^
0 restraints	Δρ_min_ = −0.56 e Å^−^^3^

### Special details

**Table:**

Experimental. The crystal was placed in the cold stream of an Oxford Cryosystems Cobra open-flow nitrogen cryostat (Cosier & Glazer, 1986) operating at 100.0 (1) K.
Geometry. All esds (except the esd in the dihedral angle between two l.s. planes) are estimated using the full covariance matrix. The cell esds are taken into account individually in the estimation of esds in distances, angles and torsion angles; correlations between esds in cell parameters are only used when they are defined by crystal symmetry. An approximate (isotropic) treatment of cell esds is used for estimating esds involving l.s. planes.
Refinement. Refinement of F^2^ against ALL reflections. The weighted R-factor wR and goodness of fit S are based on F^2^, conventional R-factors R are based on F, with F set to zero for negative F^2^. The threshold expression of F^2^ > 2sigma(F^2^) is used only for calculating R-factors(gt) etc. and is not relevant to the choice of reflections for refinement. R-factors based on F^2^ are statistically about twice as large as those based on F, and R- factors based on ALL data will be even larger.

### Fractional atomic coordinates and isotropic or equivalent isotropic displacement parameters (Å^2^)

**Table:**

	*x*	*y*	*z*	*U*_iso_*/*U*_eq_	
O1	0.66045 (19)	0.25805 (10)	0.59540 (7)	0.0188 (3)	
O2	0.68611 (19)	0.08432 (10)	0.71952 (7)	0.0175 (3)	
O3	0.80545 (19)	−0.23713 (10)	0.52116 (7)	0.0179 (3)	
N1	0.9833 (2)	−0.28189 (12)	0.22686 (9)	0.0161 (3)	
C1	1.0348 (3)	−0.30052 (16)	0.14228 (11)	0.0190 (3)	
H1A	1.0992	−0.3781	0.1266	0.023*	
C2	0.9938 (3)	−0.20721 (16)	0.07975 (11)	0.0206 (4)	
H2A	1.0305	−0.2204	0.0220	0.025*	
C3	0.8952 (3)	−0.09151 (16)	0.10463 (11)	0.0204 (4)	
H3A	0.8652	−0.0269	0.0632	0.024*	
C4	0.8425 (3)	−0.07334 (15)	0.19030 (11)	0.0180 (3)	
H4A	0.7758	0.0035	0.2063	0.022*	
C5	0.8881 (2)	−0.16930 (15)	0.25392 (11)	0.0162 (3)	
C6	0.8438 (3)	−0.15693 (15)	0.34518 (11)	0.0170 (3)	
H6A	0.8501	−0.2293	0.3822	0.020*	
C7	0.7939 (2)	−0.04592 (15)	0.37962 (10)	0.0156 (3)	
H7A	0.7785	0.0241	0.3408	0.019*	
C8	0.7615 (2)	−0.02274 (14)	0.46947 (10)	0.0146 (3)	
C9	0.7239 (2)	0.10386 (14)	0.48848 (10)	0.0151 (3)	
H9A	0.7169	0.1661	0.4435	0.018*	
C10	0.6973 (2)	0.13751 (14)	0.57154 (10)	0.0145 (3)	
C11	0.7094 (2)	0.04381 (14)	0.63954 (10)	0.0146 (3)	
C12	0.7439 (2)	−0.08119 (14)	0.62344 (10)	0.0157 (3)	
H12A	0.7494	−0.1428	0.6688	0.019*	
C13	0.7702 (2)	−0.11436 (14)	0.53926 (11)	0.0147 (3)	
C14	1.0358 (3)	−0.38729 (15)	0.29003 (11)	0.0205 (4)	
H14A	1.1044	−0.4574	0.2604	0.031*	
H14B	0.9164	−0.4098	0.3242	0.031*	
H14C	1.1208	−0.3631	0.3266	0.031*	
C15	0.6289 (3)	0.35323 (15)	0.52829 (11)	0.0229 (4)	
H15A	0.5949	0.4327	0.5519	0.034*	
H15B	0.5219	0.3379	0.4997	0.034*	
H15C	0.7485	0.3535	0.4879	0.034*	
C16	0.7042 (3)	−0.00944 (15)	0.79006 (10)	0.0182 (3)	
H16A	0.6872	0.0300	0.8427	0.027*	
H16B	0.8338	−0.0583	0.7821	0.027*	
H16C	0.6036	−0.0627	0.7925	0.027*	
C17	0.8118 (3)	−0.33027 (15)	0.59227 (11)	0.0205 (4)	
H17A	0.8410	−0.4116	0.5713	0.031*	
H17B	0.6849	−0.3229	0.6280	0.031*	
H17C	0.9137	−0.3187	0.6251	0.031*	
S1	0.74248 (6)	0.29952 (4)	0.22553 (3)	0.01886 (12)	
O4	0.7780 (2)	0.31567 (12)	0.31164 (9)	0.0316 (3)	
O5	0.9172 (2)	0.24169 (11)	0.17148 (9)	0.0286 (3)	
O6	0.5690 (2)	0.23666 (11)	0.22522 (9)	0.0247 (3)	
O7	0.49664 (19)	0.80352 (10)	0.06333 (8)	0.0205 (3)	
C18	0.6572 (3)	0.59021 (15)	0.04644 (11)	0.0181 (3)	
H18A	0.6807	0.6042	−0.0130	0.022*	
C19	0.7175 (3)	0.47361 (15)	0.08730 (11)	0.0181 (3)	
H19A	0.7829	0.4095	0.0546	0.022*	
C20	0.6816 (2)	0.45156 (14)	0.17576 (11)	0.0161 (3)	
C21	0.5851 (3)	0.54790 (15)	0.22507 (11)	0.0178 (3)	
H21A	0.5607	0.5335	0.2845	0.021*	
C22	0.5252 (3)	0.66499 (15)	0.18597 (11)	0.0179 (3)	
H22A	0.4620	0.7293	0.2189	0.021*	
C23	0.5608 (3)	0.68533 (14)	0.09672 (11)	0.0160 (3)	
C24	0.5288 (3)	0.82721 (16)	−0.02806 (11)	0.0248 (4)	
H24A	0.4792	0.9127	−0.0437	0.037*	
H24B	0.4596	0.7735	−0.0536	0.037*	
H24C	0.6690	0.8113	−0.0482	0.037*	
O1W	0.2248 (2)	0.38849 (12)	0.16774 (9)	0.0228 (3)	
H2W1	0.320 (5)	0.349 (3)	0.1858 (17)	0.047 (8)*	
H1W1	0.134 (4)	0.342 (3)	0.1731 (16)	0.043 (7)*	

### Atomic displacement parameters (Å^2^)

**Table:**

	*U*^11^	*U*^22^	*U*^33^	*U*^12^	*U*^13^	*U*^23^
O1	0.0289 (7)	0.0070 (5)	0.0203 (6)	0.0022 (5)	−0.0065 (5)	−0.0025 (4)
O2	0.0255 (7)	0.0094 (5)	0.0168 (5)	0.0022 (5)	−0.0041 (5)	−0.0017 (4)
O3	0.0261 (7)	0.0076 (5)	0.0194 (6)	0.0010 (5)	−0.0035 (5)	−0.0027 (4)
N1	0.0164 (7)	0.0121 (6)	0.0201 (7)	0.0002 (5)	−0.0046 (5)	−0.0038 (5)
C1	0.0172 (8)	0.0170 (8)	0.0234 (8)	−0.0001 (6)	−0.0026 (7)	−0.0083 (6)
C2	0.0195 (9)	0.0226 (8)	0.0197 (8)	−0.0011 (7)	−0.0023 (7)	−0.0057 (7)
C3	0.0215 (9)	0.0173 (8)	0.0222 (8)	−0.0011 (7)	−0.0049 (7)	−0.0005 (6)
C4	0.0179 (8)	0.0130 (7)	0.0233 (8)	−0.0002 (6)	−0.0043 (7)	−0.0037 (6)
C5	0.0143 (8)	0.0131 (7)	0.0223 (8)	−0.0014 (6)	−0.0039 (6)	−0.0051 (6)
C6	0.0176 (8)	0.0133 (7)	0.0198 (8)	0.0000 (6)	−0.0030 (6)	−0.0019 (6)
C7	0.0143 (8)	0.0132 (7)	0.0189 (7)	0.0003 (6)	−0.0029 (6)	−0.0023 (6)
C8	0.0127 (8)	0.0122 (7)	0.0191 (8)	0.0004 (6)	−0.0031 (6)	−0.0034 (6)
C9	0.0151 (8)	0.0102 (7)	0.0191 (7)	0.0001 (6)	−0.0027 (6)	0.0000 (6)
C10	0.0139 (8)	0.0082 (7)	0.0212 (8)	0.0018 (6)	−0.0037 (6)	−0.0034 (6)
C11	0.0140 (8)	0.0114 (7)	0.0179 (7)	0.0020 (6)	−0.0023 (6)	−0.0037 (6)
C12	0.0177 (8)	0.0099 (7)	0.0187 (8)	0.0003 (6)	−0.0032 (6)	−0.0003 (6)
C13	0.0130 (8)	0.0076 (7)	0.0234 (8)	0.0008 (6)	−0.0026 (6)	−0.0037 (6)
C14	0.0265 (10)	0.0130 (7)	0.0211 (8)	0.0038 (7)	−0.0058 (7)	−0.0028 (6)
C15	0.0365 (11)	0.0087 (7)	0.0243 (8)	−0.0006 (7)	−0.0105 (8)	−0.0002 (6)
C16	0.0240 (9)	0.0128 (7)	0.0171 (7)	0.0008 (6)	−0.0046 (7)	−0.0007 (6)
C17	0.0281 (10)	0.0094 (7)	0.0237 (8)	0.0011 (6)	−0.0068 (7)	−0.0005 (6)
S1	0.0190 (2)	0.01144 (19)	0.0263 (2)	−0.00110 (15)	−0.00810 (17)	0.00317 (15)
O4	0.0422 (9)	0.0235 (7)	0.0317 (7)	−0.0056 (6)	−0.0193 (6)	0.0066 (5)
O5	0.0217 (7)	0.0145 (6)	0.0458 (8)	0.0037 (5)	−0.0020 (6)	0.0016 (5)
O6	0.0238 (7)	0.0155 (6)	0.0354 (7)	−0.0045 (5)	−0.0081 (6)	0.0038 (5)
O7	0.0276 (7)	0.0102 (5)	0.0219 (6)	0.0033 (5)	−0.0037 (5)	−0.0008 (4)
C18	0.0233 (9)	0.0126 (7)	0.0182 (7)	−0.0006 (6)	−0.0029 (7)	−0.0027 (6)
C19	0.0211 (9)	0.0113 (7)	0.0221 (8)	0.0002 (6)	−0.0034 (7)	−0.0052 (6)
C20	0.0149 (8)	0.0111 (7)	0.0230 (8)	−0.0015 (6)	−0.0057 (6)	−0.0009 (6)
C21	0.0186 (9)	0.0164 (8)	0.0185 (8)	−0.0024 (6)	−0.0024 (6)	−0.0018 (6)
C22	0.0187 (8)	0.0132 (7)	0.0214 (8)	0.0009 (6)	−0.0023 (7)	−0.0052 (6)
C23	0.0167 (8)	0.0089 (7)	0.0223 (8)	0.0000 (6)	−0.0053 (6)	−0.0001 (6)
C24	0.0334 (11)	0.0155 (8)	0.0234 (9)	0.0041 (7)	−0.0067 (8)	0.0016 (7)
O1W	0.0200 (7)	0.0185 (6)	0.0297 (7)	0.0000 (6)	−0.0035 (6)	−0.0046 (5)

### Geometric parameters (Å, °)

**Table:**

O1—C10	1.3789 (18)	C14—H14C	0.9600
O1—C15	1.4194 (19)	C15—H15A	0.9600
O2—C11	1.3620 (19)	C15—H15B	0.9600
O2—C16	1.4366 (19)	C15—H15C	0.9600
O3—C13	1.3736 (18)	C16—H16A	0.9600
O3—C17	1.4335 (19)	C16—H16B	0.9600
N1—C1	1.359 (2)	C16—H16C	0.9600
N1—C5	1.375 (2)	C17—H17A	0.9600
N1—C14	1.477 (2)	C17—H17B	0.9600
C1—C2	1.368 (2)	C17—H17C	0.9600
C1—H1A	0.9300	S1—O4	1.4518 (14)
C2—C3	1.400 (2)	S1—O6	1.4602 (13)
C2—H2A	0.9300	S1—O5	1.4603 (14)
C3—C4	1.376 (2)	S1—C20	1.7730 (16)
C3—H3A	0.9300	O7—C23	1.3710 (18)
C4—C5	1.405 (2)	O7—C24	1.431 (2)
C4—H4A	0.9300	C18—C23	1.395 (2)
C5—C6	1.446 (2)	C18—C19	1.395 (2)
C6—C7	1.352 (2)	C18—H18A	0.9300
C6—H6A	0.9300	C19—C20	1.385 (2)
C7—C8	1.448 (2)	C19—H19A	0.9300
C7—H7A	0.9300	C20—C21	1.396 (2)
C8—C13	1.409 (2)	C21—C22	1.387 (2)
C8—C9	1.418 (2)	C21—H21A	0.9300
C9—C10	1.379 (2)	C22—C23	1.396 (2)
C9—H9A	0.9300	C22—H22A	0.9300
C10—C11	1.405 (2)	C24—H24A	0.9600
C11—C12	1.389 (2)	C24—H24B	0.9600
C12—C13	1.394 (2)	C24—H24C	0.9600
C12—H12A	0.9300	O1W—H2W1	0.80 (3)
C14—H14A	0.9600	O1W—H1W1	0.84 (3)
C14—H14B	0.9600		
			
C10—O1—C15	115.80 (12)	O1—C15—H15A	109.5
C11—O2—C16	116.97 (12)	O1—C15—H15B	109.5
C13—O3—C17	117.46 (12)	H15A—C15—H15B	109.5
C1—N1—C5	122.06 (14)	O1—C15—H15C	109.5
C1—N1—C14	117.55 (13)	H15A—C15—H15C	109.5
C5—N1—C14	120.39 (13)	H15B—C15—H15C	109.5
N1—C1—C2	121.19 (15)	O2—C16—H16A	109.5
N1—C1—H1A	119.4	O2—C16—H16B	109.5
C2—C1—H1A	119.4	H16A—C16—H16B	109.5
C1—C2—C3	118.52 (15)	O2—C16—H16C	109.5
C1—C2—H2A	120.7	H16A—C16—H16C	109.5
C3—C2—H2A	120.7	H16B—C16—H16C	109.5
C4—C3—C2	120.05 (16)	O3—C17—H17A	109.5
C4—C3—H3A	120.0	O3—C17—H17B	109.5
C2—C3—H3A	120.0	H17A—C17—H17B	109.5
C3—C4—C5	120.90 (15)	O3—C17—H17C	109.5
C3—C4—H4A	119.5	H17A—C17—H17C	109.5
C5—C4—H4A	119.5	H17B—C17—H17C	109.5
N1—C5—C4	117.27 (14)	O4—S1—O6	112.62 (8)
N1—C5—C6	118.25 (14)	O4—S1—O5	114.15 (9)
C4—C5—C6	124.48 (14)	O6—S1—O5	111.56 (8)
C7—C6—C5	123.58 (15)	O4—S1—C20	106.30 (8)
C7—C6—H6A	118.2	O6—S1—C20	105.60 (7)
C5—C6—H6A	118.2	O5—S1—C20	105.85 (8)
C6—C7—C8	127.96 (15)	C23—O7—C24	117.05 (13)
C6—C7—H7A	116.0	C23—C18—C19	118.51 (15)
C8—C7—H7A	116.0	C23—C18—H18A	120.7
C13—C8—C9	117.33 (14)	C19—C18—H18A	120.7
C13—C8—C7	125.88 (14)	C20—C19—C18	121.16 (15)
C9—C8—C7	116.76 (14)	C20—C19—H19A	119.4
C10—C9—C8	122.01 (14)	C18—C19—H19A	119.4
C10—C9—H9A	119.0	C19—C20—C21	119.53 (15)
C8—C9—H9A	119.0	C19—C20—S1	120.26 (12)
O1—C10—C9	125.62 (14)	C21—C20—S1	120.06 (13)
O1—C10—C11	115.21 (13)	C22—C21—C20	120.38 (15)
C9—C10—C11	119.17 (14)	C22—C21—H21A	119.8
O2—C11—C12	123.81 (14)	C20—C21—H21A	119.8
O2—C11—C10	115.77 (13)	C21—C22—C23	119.36 (15)
C12—C11—C10	120.41 (14)	C21—C22—H22A	120.3
C11—C12—C13	120.01 (14)	C23—C22—H22A	120.3
C11—C12—H12A	120.0	O7—C23—C18	123.36 (15)
C13—C12—H12A	120.0	O7—C23—C22	115.59 (14)
O3—C13—C12	121.44 (14)	C18—C23—C22	121.05 (15)
O3—C13—C8	117.52 (14)	O7—C24—H24A	109.5
C12—C13—C8	121.04 (14)	O7—C24—H24B	109.5
N1—C14—H14A	109.5	H24A—C24—H24B	109.5
N1—C14—H14B	109.5	O7—C24—H24C	109.5
H14A—C14—H14B	109.5	H24A—C24—H24C	109.5
N1—C14—H14C	109.5	H24B—C24—H24C	109.5
H14A—C14—H14C	109.5	H2W1—O1W—H1W1	109 (3)
H14B—C14—H14C	109.5		
			
C5—N1—C1—C2	0.0 (3)	O2—C11—C12—C13	−178.62 (15)
C14—N1—C1—C2	−179.20 (16)	C10—C11—C12—C13	1.1 (3)
N1—C1—C2—C3	−0.6 (3)	C17—O3—C13—C12	0.7 (2)
C1—C2—C3—C4	0.3 (3)	C17—O3—C13—C8	−179.24 (14)
C2—C3—C4—C5	0.7 (3)	C11—C12—C13—O3	179.71 (15)
C1—N1—C5—C4	0.9 (2)	C11—C12—C13—C8	−0.3 (3)
C14—N1—C5—C4	−179.92 (15)	C9—C8—C13—O3	179.62 (14)
C1—N1—C5—C6	−178.33 (15)	C7—C8—C13—O3	−2.5 (2)
C14—N1—C5—C6	0.8 (2)	C9—C8—C13—C12	−0.4 (2)
C3—C4—C5—N1	−1.2 (2)	C7—C8—C13—C12	177.53 (16)
C3—C4—C5—C6	177.98 (16)	C23—C18—C19—C20	0.5 (3)
N1—C5—C6—C7	164.26 (17)	C18—C19—C20—C21	−0.6 (3)
C4—C5—C6—C7	−14.9 (3)	C18—C19—C20—S1	175.15 (13)
C5—C6—C7—C8	−174.98 (16)	O4—S1—C20—C19	153.77 (14)
C6—C7—C8—C13	−2.2 (3)	O6—S1—C20—C19	−86.39 (15)
C6—C7—C8—C9	175.70 (17)	O5—S1—C20—C19	32.02 (16)
C13—C8—C9—C10	0.2 (2)	O4—S1—C20—C21	−30.55 (17)
C7—C8—C9—C10	−177.85 (15)	O6—S1—C20—C21	89.30 (15)
C15—O1—C10—C9	6.4 (2)	O5—S1—C20—C21	−152.29 (14)
C15—O1—C10—C11	−174.21 (15)	C19—C20—C21—C22	0.0 (3)
C8—C9—C10—O1	179.91 (15)	S1—C20—C21—C22	−175.73 (13)
C8—C9—C10—C11	0.6 (3)	C20—C21—C22—C23	0.6 (3)
C16—O2—C11—C12	1.7 (2)	C24—O7—C23—C18	1.2 (2)
C16—O2—C11—C10	−178.06 (14)	C24—O7—C23—C22	−179.31 (15)
O1—C10—C11—O2	−0.9 (2)	C19—C18—C23—O7	179.53 (15)
C9—C10—C11—O2	178.52 (15)	C19—C18—C23—C22	0.1 (3)
O1—C10—C11—C12	179.34 (15)	C21—C22—C23—O7	179.87 (15)
C9—C10—C11—C12	−1.2 (2)	C21—C22—C23—C18	−0.6 (3)

### Hydrogen-bond geometry (Å, °)

**Table:**

*D*—H···*A*	*D*—H	H···*A*	*D*···*A*	*D*—H···*A*
O1W—H2W1···O6	0.80 (3)	2.08 (3)	2.8703 (19)	175 (3)
O1W—H1W1···O5^i^	0.85 (3)	1.98 (3)	2.8233 (19)	173 (2)
C9—H9A···O4	0.93	2.53	3.445 (2)	167
C14—H14A···O1W^ii^	0.96	2.32	3.262 (2)	168
C14—H14C···O1^iii^	0.96	2.54	3.487 (2)	168
C16—H16A···O7^iv^	0.96	2.53	3.388 (2)	149
C16—H16B···O5^iii^	0.96	2.54	3.408 (2)	150
C16—H16C···O6^v^	0.96	2.44	3.371 (2)	163
C17—H17C···O4^iii^	0.96	2.47	3.419 (2)	168
C18—H18A···O1W^vi^	0.93	2.43	3.354 (2)	171
C22—H22A···O2^iv^	0.93	2.36	3.281 (2)	170

Symmetry codes:  (i) *x*−1, *y*, *z*; (ii) *x*+1, *y*−1, *z*; (iii) −*x*+2, −*y*, −*z*+1; (iv) −*x*+1, −*y*+1, −*z*+1; (v) −*x*+1, −*y*, −*z*+1; (vi) −*x*+1, −*y*+1, −*z*.
